# Innovation and enculturation in child communication: a cross-sectional study

**DOI:** 10.1017/ehs.2020.57

**Published:** 2020-11-09

**Authors:** C.J. Lister, B. Walker, N. Fay

**Affiliations:** School of Psychological Science, University of Western Australia, Crawley, WA 6009, Australia

**Keywords:** Sign innovation, enculturation, language development, gesture, iconicity, experimental semiotics

## Abstract

How can people achieve successful communication when using novel signs? Previous studies show that iconic signs (i.e. signs that directly resemble their referent) enhance communication success. In this paper, we test if enculturated signs (i.e. signs informed by interlocutors’ shared culture) also enhance communication success. Children, who have spent less time in their linguistic community, have less cultural knowledge to inform their sign innovation. A natural prediction is that younger children's signs will be less enculturated, more diverse and less successful compared with older children and adults. We examined sign innovation in children aged between 6 and 12 years (*N* = 54) and adults (*N* = 18). Sign enculturation, diversity and iconicity were rated. As predicted, younger children innovated less enculturated and more diverse signs, and communicated less successfully than older children and adults. Sign enculturation and iconicity uniquely contributed to communication success. This is the first study to demonstrate that enculturated signs enhance communication.

## Graphical abstract

**Figure:**
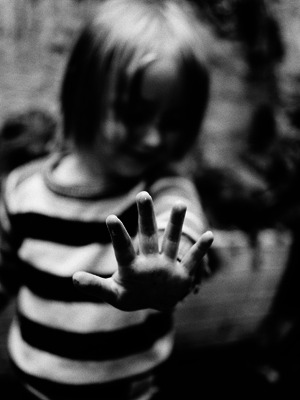

**Media summary:** Culture changes how children innovate language. Enculturation leads older children to innovate more adult-like, and more successful, sign systems.

## 1. Introduction

Language is a complex, adaptive system; new languages are created, and old languages evolve and adapt to their contextual niche (Beckner et al., 2009). For instance, the meaning of many historical texts is ambiguous to modern readers (e.g. the adjective ‘nice’ originally described an ignorant or ‘simple’ person; while in Chinese hanzi, the original character for mountain; 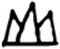 has evolved into 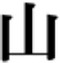; Vaccari & Vaccari, 1961). Modern languages frequently change with the addition of new words and the loss old words (e.g. recent updates to the Oxford English dictionary saw the addition of ‘cryptocurrency’, ‘Jedi’ and ‘whatevs’ while updates to the *Merriam–Webster Collegiate Dictionary* saw the removal of the obsolete words ‘frutescent’ and ‘snollygoster’; OED, n.d.; Merriam–Webster, 1941).

The flexibility of human communication is illustrated by people's ability to create ad hoc language systems. For example, at a Nicaraguan school for the deaf, children with no common language rapidly innovated a novel sign language. As new cohorts of children joined the school, adding their own linguistic innovations, the gestural communication system took on the grammatical and structural properties of a fully fledged sign language (Senghas & Coppola, 2001; Goldin-Meadow, 2010). Likewise, pidgins rapidly emerge among groups of people who do not share a common language. Over time, and with children in the community using them as their primary languages, pidgins develop into creoles: fully fledged languages (Velupillai, 2015). Linguistic innovation and evolution have also been examined under laboratory conditions. In these experiments, participants try to innovate new labels for objects or concepts without using their shared language. These experiments show that adults (e.g. Fay, Arbib & Garrod, 2013; Garrod, Fay, Lee, Oberlander & MacLeod, 2007) and children (e.g. Bohn, Kachel & Tomasello, 2019; Lister, Burtenshaw, Walker, Ohan & Fay, under review) can innovate novel signs from scratch, and that these signs become increasingly efficient with repeated use. So, a key feature of language is its flexibility, which in turn enables people to innovate novel signs and systems of communication as needed.

Language use is a joint activity that occurs between people, the goal of which is cognitive alignment (e.g. Fusaroli, Rączaszek-Leonardi & Tylén, 2014; Clark & Wilkes-Gibbs, 1986; Garrod & Pickering, 2004; 2009, Lister & Fay, 2017). Cognitive alignment is enabled by behaviour alignment. When interlocutors align their behaviour (i.e. when they use the same sign to communicate the same meaning), communication is more successful (Fay, Walker, Swoboda & Garrod, 2018). In natural language studies, interlocutors align their behaviour at several levels; for example, at prosodic (Pardo, 2006), lexical (Brennan & Clark, 1996; Branigan, Pickering, Pearson, McLean, & Brown, 2011) and syntactic levels (Branigan, Pickering, McLean, & Cleland, 2007). This interactive alignment process is what makes conversation easy (Garrod & Pickering, 2004). Local alignment also scales up to larger groups, giving rise to population-level global alignment (Barr, 2004; Fay, Garrod, Roberts & Swoboda, 2010).

So far, we have highlighted the role of innovation in language creation and evolution, and the importance of linguistic alignment to successful communication. However, these processes give rise to a tension: someone who innovates a new sign is – by definition – not aligned with the other members of their linguistic community. This tension between sign innovation and alignment can be resolved if people innovate signs that are *salient* to members of their community. Salient signs are focal (or Schelling) points: solutions that members of the same community tend to choose by default in the absence of communication (Schelling, 1960). This solution-alignment enables people to coordinate in the absence of direct interaction. A well-known example is Schelling's New York City problem (Schelling, 1960): two strangers play a coordination game that requires them to meet in New York City but does not allow them to communicate. When participants (all from New Haven, Connecticut) were posed with this problem, they were disproportionately likely to select noon at the information booth at Grand Central Station. Because the same solution was salient to participants, their choices were aligned, and they solved the coordination problem. The same solution was salient to participants because they shared a common culture. In the context of communication, two strangers who live in the same town may both use the phrase ‘the hill’ to refer to the same location: a reference point that may not be salient to an out-of-towner (who may think ‘the hill’ means a different hill, or the name of a pub, for instance). This suggests that enculturation (the process by which a person aligns with the culture of those around them) will be critical to innovating salient solutions that enable successful coordination.

Linguistic enculturation is evident in the earliest stages of language development. For example, infants’ babbling is initially acoustically similar across different cultures, but over time, the phonemes they produce align with their linguistic community (e.g. Boysson-Bardies, Sagart & Durand, 1984; Lee, Davis & Macneilage, 2010). This process of enculturation occurs across other aspects of language, including the lexicon and syntax (Slobin, 1990; Harkins, Koch & Michel, 1994). Enculturation occurs gradually (Shimahara, 1970) and children (who have spent less time in their linguistic community) are less enculturated than adults. Lower levels of enculturation among children may cause them to innovate signs that are less aligned with their community. This may help explain why children are prolific innovators: they innovate more diverse solutions to problems because their solutions are not constrained, guided or informed, by pre-existing cultural conventions (Gopnik, Griffiths & Lucas, 2015).

As children age, they become increasingly enculturated. Hypothesis 1 is that, compared with younger children, older children will innovate signs that are more similar to those innovated by enculturated adult members of their linguistic community. Lower enculturation among younger children may lead them to innovate more diverse signs. Hypothesis 2 is that younger children will innovate a more diverse range of signs compared with older children and adults. Lower enculturation among children, and their (related) tendency to innovate diverse solutions to problems, makes them ideal candidates to study the relationship between enculturation and communication success.

Experimental semiotic tasks offer a method to study sign innovation under controlled laboratory conditions. These tasks examine how people innovate novel signs when prohibited from using conventional language, often in a different modality, e.g. through movement (Scott-Phillips, Kirby & Ritchie, 2009), drawing (Garrod et al., 2007; Garrod, Fay, Rogers, Walker & Swoboda, 2010; Caldwell & Smith, 2012), non-lexical vocalization (Perlman, Dale & Lupyan, 2014, 2015; Perlman & Lupyan, 2018) or gesture (Fay et al., 2013; Fay, Ellison & Garrod, 2014a; Lister, 2019). Much of this research has examined a core aspect of sign salience–iconicity: the degree to which a sign resembles its meaning. For example, in Fay et al. (2013) and Fay, Lister, Ellison and Goldin-Meadow (2014b) participants tried to communicate a list of words to a partner using either gesture or non-lexical vocalization. Communication success (i.e. the percentage of words their partner correctly guessed) was higher in the gesture modality than the vocal modality. The authors speculated that improved communication success in the gesture modality resulted from the greater affordance for iconic sign innovation in this modality compared with the vocal modality. Using a similar paradigm, Lister (2019) measured sign iconicity directly, by rating how closely each sign resembled its meaning. They found that sign iconicity was greater in the gesture modality than the vocal modality, and that greater iconicity was associated with greater communication success. Thus, communication success was enhanced by the use of signs that were salient to participants by virtue of their iconicity.

Enculturation may also contribute to sign salience. Lister et al. (under review) studied sign creation in children using an experimental semiotic task. Children aged 6–12 years innovated novel gestures and vocalizations to communicate a list of words. Recordings of the children's signs were later played to adult interpreters who tried to guess each sign's meaning. A second cohort of adult participants rated the iconicity of the children's signs. Children's ability to communicate successfully improved with age, and children innovated more iconic signs and communicated more successfully through gesture than through non-linguistic vocalization, replicating Fay et al. (2013, 2014b). However, sign iconicity did not fully explain children's communication success: despite producing signs that scored similarly on iconicity, 8- and 9-year old children had lower communication success than 10- to 12-year old children.

Lister et al. (under review) suggested that older children's greater enculturation may have contributed to their improved communication success relative to the younger children. For instance, younger children used their hands to simulate the movement of clock hands to communicate ‘time’ (a sign that directly resembles a clock), while older children, like adults, tapped their wrist to indicate a watch (a sign that is consistent with enculturation in a society with wrist watches; see example videos at: https://osf.io/jtng2/). To our knowledge, no study has tested if enculturation contributes to communication success. Moreover, we do not know if the ability to innovate culturally salient signs develops in children as they become increasingly enculturated. The present paper examines the relationship between sign iconicity, enculturation and communication success, and tests whether participants of different ages (6–7 years, 8–9 years, 10–12 years and 18+ years) can innovate culturally salient signs to achieve successful communication.

## 2. The present study

The present study uses a combination of pre-existing data (communication success and iconicity ratings from Lister et al., under review) and new data (generated by coding the enculturation/diversity of the gestures and vocalizations produced by participants in Lister et al., under review) to test three hypotheses, presented in Table 1.

**Table 1.** Summary of Hypotheses 1–3 and their predictions

Hypothesis 1	Older children will be more enculturated than younger children. This will be reflected by greater sign similarity between adults and older children, and lower sign similarity between adults and younger children.
Hypothesis 2	Younger participants will innovate more diverse signs. This will be reflected by lower sign similarity between younger children (and their same-aged peers) compared with older children (and their same-aged peers). Adults, who have experienced the most enculturation, will innovate the least diverse signs.
Hypothesis 3	Enculturation will contribute to communication success. This will be reflected by a positive relationship between sign enculturation (operationalized as similarity to adult signs) and communication success (controlling for sign iconicity).

## 3. Method

Lister et al. (under review) filmed 18 adults and 54 children (split evenly into three age groups: ‘young’, 6–7 years; ‘middle’, 8–9 years; and ‘old’, 10–12 years) acting as Producers, innovating novel gestures and non–linguistic vocalizations to communicate meanings from a list of words (e.g. ‘lion’, ‘fun’, ‘sneeze’). Later, a second cohort of adult participants (Interpreters) viewed the Producers’ signs and attempted to guess their meanings. The authors measured participants’ communication success (operationalized as identification accuracy; whether or not the Interpreter correctly guessed the sign's meaning). A third cohort of adult participants (Iconicity Raters) viewed the Producers’ recorded signs and rated the iconicity of each sign on a seven-point Likert scale (0 = not at all iconic, 6 = highly iconic).

In the present study, we used the communication success and iconicity data from Lister et al. (under review). Child enculturation was measured by rating the similarity of child signs to adult signs. Sign diversity was measured by rating the similarity of the signs innovated by participants from within the same age group (within-group similarity). Sign similarity was measured by comparing pairs of signs; for each similarity comparison, two same-modality recordings of the same word were presented side-by-side on a computer screen and their similarity was rated on a seven-point Likert scale (0 = not at all similar, 6 = highly similar).

**Figure 1.**
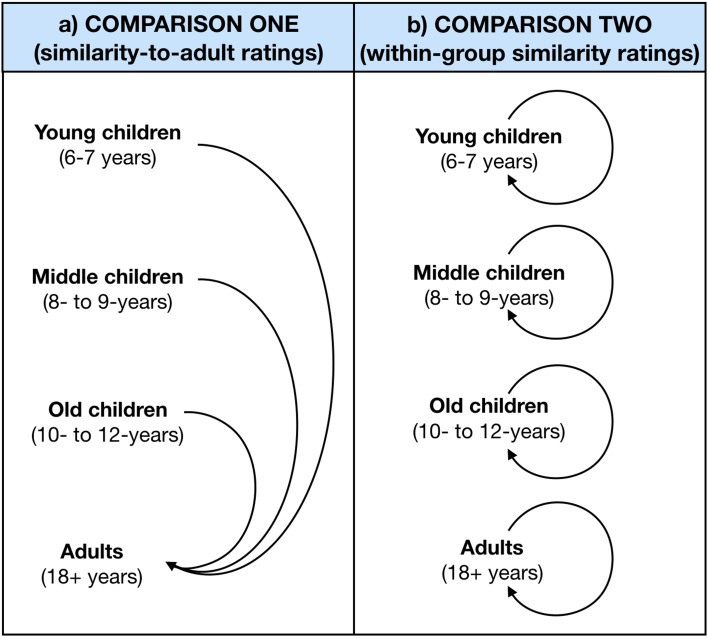
Diagram of the similarity comparisons made (a) between children in the Young (6–7 years), Middle (8–9 years) and Old (10–12 years) age groups and Adults, and (b) within each age group.

Figure 1 shows the comparisons made to assess the similarity of the signs innovated by children to those innovated by adults, and to measure within-group sign similarity. For the similarity-to-adult sign ratings (Figure 1a), the signs innovated by each child were compared with the signs that adults innovated, for the same word in the same modality. This allowed us to test whether older children's signs were more similar to adults’ signs, compared with younger children (Hypothesis 1). For the within-group similarity ratings (Figure 1b), the signs innovated by each child were compared with the signs innovated by other children in the same age group (again for the same word in the same modality). This allowed us to test whether younger children's signs were more diverse than older children's and adults’ signs (Hypothesis 2).

For each analysis, we computed a baseline measure of sign similarity. To compute a baseline, we took 20% of the original similarity comparisons and replaced one of the two constituent recordings with another recording from the replaced participant, in the same modality but communicating a different word. Baseline comparisons establish the level of sign similarity that occurs by chance between signs for unrelated meanings when controlling for participant and modality.

## 4. Results

The similarity ratings were analysed using cumulative link mixed effects modelling (a method recommended when analysing responses made on Likert scales and other ordinal datasets; McElreath, 2020). Prior to analysis, the fixed effects (Age Group and Modality) were factor coded, with Age Group coded as an ordinal factor with three levels (Young, Middle, Old). The dependent variable – Sign Similarity – was coded as an ordinal factor with seven levels (0–6). Linear mixed effects modelling was used to analyse the relationships between participants’ mean Sign Iconicity, Sign Similarity and Communication Success scores. All analyses were performed, and all figures were created, in R (R Core Team, 2019). Statistical models were estimated using the clmm() function of the ordinal package (Christensen, 2019) and the lmer() function of lme4 (Bates, Mächler, Bolker, & Walker, 2015). Model fit was compared using maximum likelihood estimation (see Supplementary Analyses). The maximal random effects structure justified by the experiment design was specified where possible (Barr, Levy, Scheepers, & Tily, 2013). The data, full model specifications and R Script are available on the Open Science Framework: https://osf.io/jtng2/.

In Section 4.1, we test Hypothesis 1: that older children will be more enculturated than younger children. This would be reflected by greater sign similarity between adults and older children, and lower sign similarity between adults and younger children. In Section 4.2, we test Hypothesis 2: that younger children will innovate more diverse signs than older children. This would be reflected by lower sign similarity between younger children (and their same-aged peers) compared with older children (and their same-aged peers). Adults, who have experienced the most enculturation, would innovate the least diverse signs. In Section 4.3, we test Hypothesis 3: that enculturation will contribute to communication success. This would be reflected by a positive relationship between sign enculturation (operationalized as similarity to adult signs) and communication success (controlling for sign iconicity).

### 4.1. Enculturation increases with age

**Figure 2.**
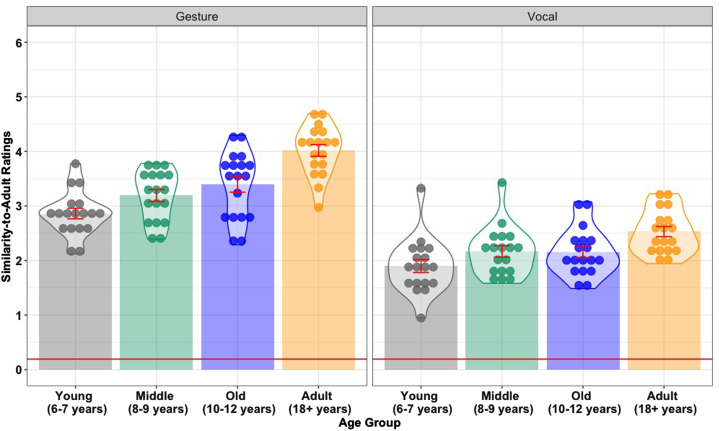
Mean sign similarity between adults and children in the Young (6–7 years), Middle (8–9 years) and Old (10–12 years) age groups, and mean sign similarity within the Adult (18+ years) group, in the gesture and vocal modalities. Each data point represents a participant's average similarity to the adult cohort. Error bars represent 95% confidence intervals. The horizontal red line indicates baseline sign similarity.

Hypothesis 1 predicts that older children will be more enculturated than younger children. The best-fitting model included fixed effects for Modality (*β* = −0.86, SE = 0.05, *z* = −18.12, *p* < 0.001) and Age Group (*β* = 0.47, SE = 0.05, *z* = 9.76, *p* < 0.001). The Age Group effect was driven by lower similarity to adult signs in the Young age group (6–7 year-olds) compared with the Middle group (8–9 year-olds) (*β* = 0.17, SE = 0.04, *z* = 3.72, *p* < 0.001), and in the Old age group (10–12 year-olds) compared with the Adults (*β* = 0.23, SE = 0.05, *z* = 4.79, *p* < 0.001). There was no statistical evidence of a difference between the Middle and Old age groups (*β* = 0.04, SE = 0.04, *z* = 1.02, *p* = 0.310). This pattern of results supports Hypothesis 1. In addition, participants’ gestures were more similar to those of their linguistic community than their non-lexical vocalizations. This suggests that gesture offers more scope for enculturated sign innovation than vocalization (see Figure 2).

### 4.2. Younger children innovate more diverse signs

**Figure 3.**
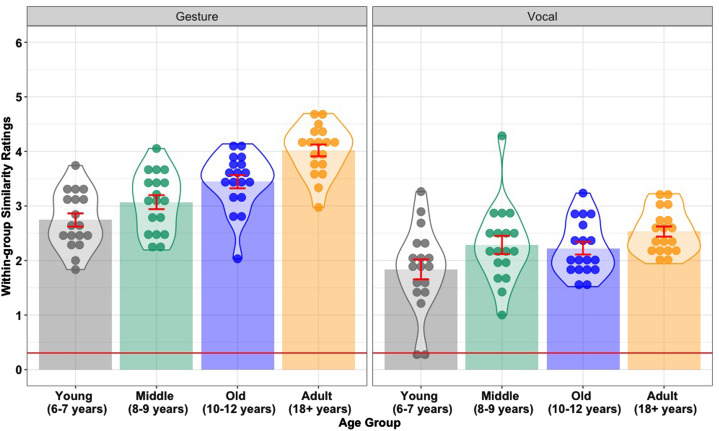
Mean sign similarity within age groups in the gesture and vocal modalities, across Young (6–7), Middle (8–9), Old (10–12) and Adult (18+) age groups. Each data point represents a participant's average sign similarity to the other participants in their age group (for the same word communicated the same modality). Error bars represent 95% confidence intervals. The horizontal red line indicates baseline sign similarity.

Hypothesis 2 predicts that younger children will innovate more diverse signs than older children. The best-fitting model included fixed effects for Modality (*β* = −0.82, SE = 0.06, *z* = −14.15, *p* < 0.001) and Age Group (*β* = 0.52, SE = 0.08, *z* = 6.94, *p* < 0.001). The Age Group effect was driven by lower within-group sign similarity in the Young age group (6–7 year-olds) compared with the Middle group (8–9 year-olds) (*β* = 0.18, SE = 0.09, *z* = 2.03, *p* = 0.042), and in the Old age group (10–12 year-olds) compared with the Adults (*β* = 0.20, SE = 0.06, *z* =3.29, *p* < 0.001). There was no statistical evidence of a difference between the Middle and Old age groups (*β* = 0.10, SE = 0.07, *z* = 1.30, *p* = 0.193). This pattern of results supports Hypothesis 2. In addition, there was more within-group diversity for participants’ non-lexical vocalizations compared with their gestures (see Figure 3).

### 4.3. Sign enculturation enhances communication success

**Figure 4.**
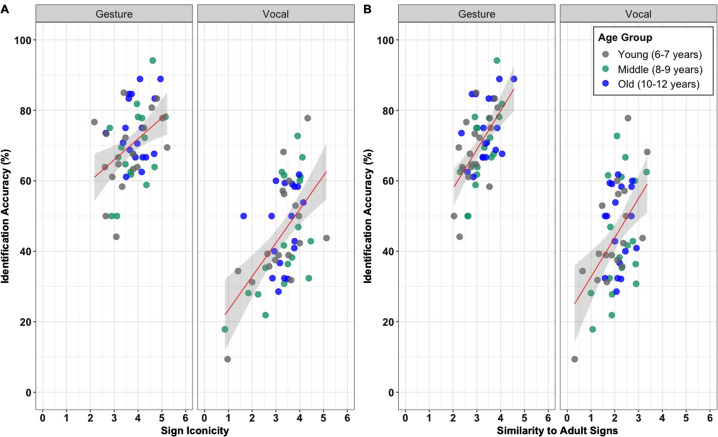
(a) Relationship between children's mean sign iconicity rating and their mean communication success (operationalized as identification accuracy – the percentage of each child's signs that were guessed correctly by the adult participants). (b) Relationship between children's mean sign enculturation rating and their mean communication success (operationalized as identification accuracy – the percentage of each child's signs that were guessed correctly by the adult participants). Each dot point represents one child participant. The linear regression line is indicated in red, shading on either side of the regression line represents a 95% confidence interval.

Hypothesis 3 predicts that enculturation will enhance communication success. This will be reflected by a positive relationship between sign enculturation (operationalized as similarity to adult signs) and communication success (controlling for sign iconicity). The best-fitting model included fixed effects for Iconicity (*β* = 0.12, SE = 0.01, *t* = 14.66, *p* < 0.001) and for Enculturation (*β* = 0.05, SE = 0.01, *t* = 4.45, *p* < 0.001). There was no statistical evidence of an interaction between Iconicity and Enculturation (*β* = −0.00, SE = 0.00, *t* = −1.42, *p* = 0.156). Note also that Iconicity and Enculturation were moderately correlated, *r*(52) = 0.52, *p* < 0.001 (gesture), *r*(52) = 0.58, p < 0.001 (vocalization), but not strongly enough to be considered collinear. As per Lister et al. (under review), greater sign iconicity was positively associated with communication success (see Figure 4a). This pattern supports Hypothesis 3, that more enculturated signs improve communication success (see Figure 4b).

## 5. Discussion

Communication systems can emerge and evolve rapidly through individual innovations, but for these systems to be effective, people must be able to align on the meaning of the innovated signs. This gives rise to a tension between sign innovation and alignment. One way to resolve this tension is through the production of iconic signs, which share a direct resemblance to their referent and so can be understood without prior knowledge (Lister & Fay, 2017). Another way to resolve this tension is through the creation of enculturated signs – signs that are mutually intelligible to members of the same linguistic community because of their shared cultural knowledge. We tested this using a cross-sectional developmental study that examined children's and adults’ ability to innovate novel signs. Recruiting child participants allowed us to examine sign innovation in a less enculturated group whose signs were therefore less likely to be informed by pre-existing cultural knowledge.

We first tested if older children were more enculturated than younger children (Hypothesis 1). As predicted, older children innovated signs that were more similar to the signs innovated by adults. This suggests that enculturation guides children to innovate signs that are aligned with members of their linguistic community. Next, we tested if younger children innovated more diverse signs than older children and adults (Hypothesis 2). As predicted, younger children's signs were more diverse (i.e. less similar to the signs innovated by their same-aged peers). Given that diversity is a corollary of enculturation, it follows that, as children become more enculturated, and their sign innovation is constrained by cultural norms, sign diversity is reduced. Last, we tested if enculturation contributes to communication success (Hypothesis 3). As predicted, sign enculturation was positively associated with communication success (controlling for sign iconicity).

### 5.1. Adaptive benefits of diversity

In the present study, lower enculturation/greater sign diversity negatively impacted communication success. However, in other contexts, diversity is advantageous. In a problem-solving task, Gopnik et al. (2015) showed that children innovated more diverse solutions and engaged in more flexible problem solving than adults. Although this reduced efficiency, children's broader search strategy led them to discover better solutions (Gopnik et al., 2015). Gopnik et al. suggest that *Homo sapiens*’ extended childhood provides a safe period for exploration and learning, enabling young children to act as ‘hypothesis testers’ – exploring a broad range of solutions while protected by caregivers. As children age, they internalize the norms of their culture, and their tendency to innovate diverse solutions is replaced by a tendency to innovate more culturally informed solutions.

The broader search strategy of young children, and the diversity it gives rise to, can have benefits at the population level. In social groups, diversity of solutions and selective social learning give rise to cumulative cultural evolution (Tomasello, 2009; Tamariz, Ellison, Barr,& Fay, 2014). This can help explain why it was children (and not adults) at the Nicaraguan school for the deaf who were primarily responsible for creating the new signed language, and why the language grew in complexity and structure with each new cohort of child learners who joined the school (Senghas & Coppola, 2001). So, although diversity did not benefit participants in the present study, there is evidence that diversity carries benefits at the population level.

### 5.2. Gesture is more enculturated and less diverse than vocalization

We found greater enculturation and lower diversity for signs innovated in the gesture modality compared with the vocal modality. A similar pattern of results was observed in a cross-cultural study comparing the efficacy of communication in the gesture and vocal modalities (Fay et al., under review). Why do people innovate more similar gestured signs than vocal signs?

A candidate explanation relates to the embodied nature of gesture. Several accounts of language innovation suggest that gestural communication systems emerge out of goal-directed actions that, over time, take on a communicative role (e.g. Glenberg & Gallese, 2012; Ortega, Schiefner & Ozyurek, 2019; Cook & Tanenhaus, 2009; Hostetter & Alibali, 2008; Kita, Alibali & Chu, 2017). In other words, the ways in which people conceptualize real-world objects or concepts derives from the ways in which they physically interact with those objects or concepts (Hostetter & Alibali, 2008). Grounding real-world concepts in terms of their associated motor schemas makes it likely that people who interact physically with the world in similar ways will tend to align on the same gestures when communicating the same concepts (Ortega et al., 2019). This may explain why we observed more similarity among participants’ gestures than their vocalizations: participants of all ages tend to interact with their environment in similar physical ways, so when asked to represent the world through gestures, they tended to align upon similar (and mutually salient) solutions.

### 5.3. Enculturation improves communication success

Lister et al. (under review) found that children who innovated iconic signs enjoyed greater communication success, and that older children innovated more iconic signs than younger children. However, sign iconicity did not fully explain why older children communicated more successfully than younger children. The authors speculated that enculturation may also contribute to older children's communication success. This was tested in the present study. As predicted, sign enculturation was positively associated with communication success. Enculturation and iconicity were related, but not strongly enough to be considered collinear. This indicates that enculturation and iconicity are separate but related attributes, and that each uniquely contributes to communication success.

It is important to recognize that correlation does not imply causation, and that other factors may explain why communication success increased with age. For instance, age-related improvements in cognitive abilities (e.g. perspective taking, Perner & Wimmer, 1985; theory of mind and executive control, Carlson & Moses, 2001; problem solving, Holyoak, Junn & Billman, 1984) may explain differences in communication success or may mediate the relationship between enculturation and communication success. While we cannot disentangle the effects of age and enculturation on communication success, we offer two plausible explanations for the observed results: a deliberative and a non-deliberative account of sign creation (see also Rogers, Fay & Maybery, 2013; Kahneman, 2011).

According to a Deliberative account, people strategically draw on cultural knowledge to inform sign innovation. So, they must not only possess relevant cultural knowledge, but must also have the cognitive abilities necessary to capitalize on that knowledge. On this account children in the current study had similar levels of enculturation, but only older children had the cognitive abilities necessary to capitalize on their cultural knowledge and use it to inform sign innovation. In contrast, on a Non-Deliberative account, people's cultural knowledge implicitly informs sign innovation. In any linguistic community, some signs will be more culturally salient than others. Enculturation in that community increases the likelihood that these signs will be more activated and so more likely to be innovated. On this account, older children, who have spent more time in their linguistic community, will innovate more enculturated signs that are more salient to that community.

While the present study cannot distinguish between a Deliberative or a Non-deliberative account, our results demonstrate that enculturated signs are positively associated with communication success. Future experiments should look to disentangle children's cognitive abilities from their capacity to innovate enculturated signs.

## 6. Conclusion

Our findings speak to the affordance of enculturated signs when innovating novel human communication systems. We demonstrate that older children innovate more enculturated signs compared with younger children and that sign enculturation enhances communication success. The importance of iconic signs when innovating language systems is well established. Less understood is the role that enculturation plays in facilitating sign innovation, and how this leads to more successful communication among members of the same linguistic community. Our results indicate that both enculturation and iconicity play an important role when innovating a communication system.

## Supplementary material

For supplementary material accompanying this paper visit http://dx.doi.org/10.1017/ehs.2020.57. click here to view supplementary material

## Research transparency and reproducibility

The data and analyses associated with this paper are available in Open Science Framework at https://osf.io/jtng2/.
